# Young, male, road traffic victims: a systematic review of the published trauma registry literature from low and middle income countries

**DOI:** 10.1051/sicotj/2015007

**Published:** 2015-06-15

**Authors:** Oliver Boughton, Gareth G. Jones, Christopher B.D. Lavy, Caris E. Grimes

**Affiliations:** 1 The MSk Lab, Imperial College London London SW7 2AZ UK; 2 Nuffield Department of Orthopaedics, Rheumatology and Musculoskeletal Sciences, Oxford University Oxford OX1 2JD UK; 3 King’s Centre for Global Health, King’s College London London WC2R 2LS UK

**Keywords:** Trauma registry, Low and middle-income countries

## Abstract

*Background*: Trauma contributes significantly to the global burden of disease. We analysed published trauma registries to assess the demographics of those most affected in low and middle-income countries (LMICs).

*Methods*: We performed a systematic review of published trauma registry studies according to PRISMA guidelines. We included published full-text articles from trauma registries in low and middle-income countries describing the demographics of trauma registry patients. Articles from military trauma registries, articles using data not principally derived from trauma registry data, articles describing patients of only one demographic (e.g. only paediatric patients), or only one mechanism of injury, trauma registry implementation papers without demographic data, review papers and conference proceedings were excluded.

*Results*: The initial search retrieved 1868 abstracts of which 1324 remained after duplicate removal. After screening the abstracts, 78 full-text articles were scrutinised for their suitability for inclusion. Twenty three papers from 14 countries, including 103,327 patients, were deemed eligible and included for analysis. The median age of trauma victims in these articles was 27 years (IQR 25–29). The median percentage of trauma victims who were male was 75 (IQR 66–84). The median percentage of road traffic injuries (RTIs) as a percentage of total injuries caused by trauma was 46 (IQR 21–71).

*Conclusions*: Young, male, road traffic victims represent a large proportion of the LMIC trauma burden. This information can inform and be used by local and national governments to implement road safety measures and other strategies aimed at reducing the injury rate in young males.

## Introduction

In 2010 there were 5.1 million worldwide deaths attributable to injury. This accounts for 9.6% of all global deaths and has been increasing over the last 20 years [18]. To put this in context, injuries account for more deaths than HIV-AIDS (human immunodeficiency virus-acquired immune deficiency syndrome), tuberculosis and malaria combined [24]. In low and middle-income countries (LMICs) there is a greater toll of injury than high-income countries [40], with 90% of world deaths resulting from injury occurring in LMICs [49]. Injury can also result in lifelong disability [49], with significant financial implications for the injured patient and their family [42]. Injuries disproportionately affect males and the young [11, 24].

As a subgroup of injuries, road traffic injuries (RTIs) are the leading injury-related cause of death in males and were the ninth leading cause of death worldwide in 1999 [31]. RTIs accounted for 14% of deaths in males aged 10–24 years and 5% of female deaths in the same age group in 2004 [30]. In 2010, RTIs accounted for 1.3 million deaths worldwide and there was a 46% increase in death due to RTIs compared to two decades earlier [18]. Whereas deaths in high-income countries with road safety programmes have reduced over the last few years, deaths from RTIs in LMICs have increased [18]. RTIs are predicted to become the third or fourth leading cause of death in the world by 2030 [19].

A trauma registry may be defined as “a timely, accurate, and comprehensive data source that allows for continuous monitoring of the process of injury care” [26]. The data encompasses all hospital trauma-related admissions and is a powerful tool for identifying injury trends and possible solutions [4, 29]. Trauma registry data are particularly valuable in LMICs because other sources of data, which might be available in high-income countries, are less accessible in LMICs [25].

We set out to use published trauma registry data from LMICs to determine the current demographics of trauma patients in LMICs, as a basis for the development of intervention strategies. Specifically, we wanted to answer the questions:

1. Do young, male patients continue to be most affected by trauma?
2. How much do RTIs contribute to the burden of trauma in LMICs?

To answer these questions we performed a systematic review of the published trauma registry literature from LMICs.

## Method

We performed a systematic review of the published trauma registry literature from LMICs. Medline, Embase, Cochrane Library, PubMed, CINAHL and Web of Science from design to the 30th May 2014 were searched using single and combinations of the search terms “developing world”, “developing country”, “low income country”, “middle income country”, “trauma database/databank”, “trauma registry/registries”, “injury database/databank” and “injury registry/registries”. We included published full-text articles from trauma registries in low and middle-income countries (as defined by the World Bank [1]) that describe the demographics of their trauma registry patients. Authors were contacted by email if full-text articles were unavailable. Articles from military trauma registries were excluded on the basis that their patient demographics and mechanisms of injury would be different. Articles from high-income countries, articles using data not principally derived from a trauma registry, articles describing patients of only one demographic (e.g. only paediatric patients) or only one mechanism of injury (e.g. only RTIs) were excluded from the final analysis. Trauma registry implementation or design papers, review papers and conference proceedings were excluded. Two authors selected articles for the qualitative and quantitative analyses and disagreements about whether a study should be included were resolved by discussion, as advised by the Cochrane Collaboration [10].

For the quantitative analysis, articles that used the same data from another article were not included. For example, if there were two articles published from the same trauma registry data of the same or similar years, only one of the articles would be chosen for quantitative analysis. During the qualitative analysis if data from an article were decided to not be trauma registry data, if the data only represented one patient group (e.g. one age group of patients) or if the data were incomplete, the article would be excluded from the quantitative analysis. Data from the included articles were analysed using IBM SPSS Statistics version 22. Average patient age, gender and mechanism of injury were analysed. Additional data on method and time of pre-hospital transfers were analysed, if available. Medians and interquartile ranges were chosen to represent the results, as the data distribution was non-parametric. If an article reported the average age of their patients as both a median and a mean, the median value was chosen for the purpose of analysis. If, however, only a mean was reported, the mean was accepted and used for the analysis. An assessment of the quality of articles was made, based on the “Trauma Registry Assessment Tool” designed by O’Reilly et al. [28].

## Results

**Figure 1.**
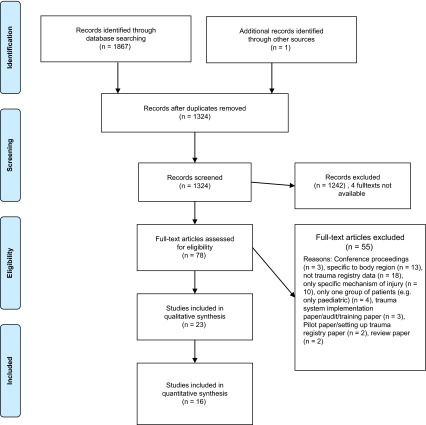
Systematic review flowchart, using PRISMA guidelines for systematic reviews.

Figure 1 shows the systematic review flowchart according to PRISMA guidelines for systematic reviews [21]. The search retrieved 1867 abstracts from database searching and one additional record from a reference in an article’s bibliography. Abstracts (1324) remained after duplicates were removed and 78 full-text articles were reviewed after abstract screening. Twenty three papers from 14 countries, including 103,327 patients, were deemed eligible and included in the qualitative analysis. Table 1 displays the articles included in the qualitative analysis. Sixteen of these articles were included in the quantitative analysis. The explanations for why seven articles were not included in the quantitative analysis are contained in the table. Table 2 displays an assessment of the quality of articles in the qualitative analysis. We made an overall subjective assessment of the quality of the articles retrieved by comparing a trauma registry article to what O’Reilly et al. recommend a trauma registry should report using their “Trauma Registry Assessment Tool” [28].

**Table 1.** Articles included in qualitative synthesis of systematic review.

Country	Author, Year	Methodology	Number of hospitals	Number of patients (over study period)	Included in quantitative analysis
Columbia	Ordóñez et al. 2012 [29]	Electronic trauma data capture in emergency department for trauma patients.	2	3923 (3 months)	Yes
Fiji	Wainiqolo et al. 2012 [43]	Paper injury surveillance form using data captured from medical notes (inpatients only).	12	2233 (1 year)	No: injury surveillance data captured from inpatient medical notes only.
India	Roy et al. 2010 [37]	Paper trauma checklist on admission to trauma ward. One hundered and seventy randomly selected patients from a total of 454 patients admitted to trauma ward. Excluded: elderly patients with an isolated fracture of the neck of the femur.	1	170 (2 months)	Yes
Iran	Haghparast-Bidgoli et al. 2013 [8]	Validated trauma questionnaire completed for all admitted trauma patients.	14	17,753 (5 years)	Yes
Iran	Moini et al. 2000 [22]	Paper trauma registry form completed for all trauma patients in emergency department and followed up daily on the ward.	3	2663 (1 year)	No: Same data as Rabbani/Moini paper.
Iran	Rabbani and Moini 2007 [34]	Paper trauma registry form completed for all trauma patients in emergency department and followed up daily on the ward.	3	4096 (likely over 7 years but not recorded)	Yes
Jamaica	Plummer et al. 2010 [33]	Electronic trauma database. Patients aged 25–29 years selected from the database.	1	715 (5 years)	No: only 25–29 year olds included.
Jamaica	Ward et al. 2010 [44]	Paper injury surveillance form for trauma patients on arrival in hospital or after stabilised if critically unwell.	9	40,563 (1 year)	Yes
Malawi	Samuel et al. 2010 [39]	Emergency department trauma registry form filled out for trauma patients on arrival and retrospective review of all hospital ward admissions, discharges and report log books. Combined data.	1	1474 (6 months)	Yes
Malaysia	Sabariah et al. 2008 [38]	All major trauma patients’ details directly entered into electronic database.	5	933 (1 year)	Yes
Nigeria	Nottidge et al. 2014 [25]	Paper trauma registry forms obtained prospectively in emergency department for all patients with injuries.	1	93 (7 weeks)	Yes
Pakistan	Hashmi et al. 2013 [9]	Computerised database of all activated trauma team calls (dead on arrival and burns excluded).	1	1227 (12 years)	Yes
Pakistan	Mehmood et al. 2013 [20]	Electronic trauma registry of all trauma patients in emergency department (excluding isolated hip fractures and dead on arrival).	1	542 (3 months)	Yes
Pakistan	Zafar et al. 2002 [51]	Initial paper trauma form for all patients meeting trauma team activation criteria, converted to online database. Patients operated on in other hospitals excluded.	1	279 (2 years)	No: same data as Hashmi et al.’s paper.
Rwanda	Petroze et al. 2014 [32]	Paper trauma registry forms completed for all injured patients transferred from a district hospital, who died in the emergency department or admitted due to injury included. Minor injuries treated as an outpatient excluded.	1	2227 (1 year)	Yes
South Africa	Laing et al. 2014 [17]	Electronic trauma registry. Inclusion criteria: all trauma-related admissions, all trauma-related mortalities. Exclusion criteria: all orthopaedic trauma cases managed without co-supervision or consultation from trauma surgeons, all trauma cases managed as outpatients, burns patients, attempted suicides by way of poison or caustic substance ingestion, foreign body ingestion, inhalation.	2	2550 (1 year)	Yes
South Africa	Schuurman et al. 2011 [40]	Paper trauma registry forms filled out for all trauma patients in emergency department.	1	785 (1 month)	Yes
Turkey	Squyer et al. 2008 [42]	Medical records of all trauma patients admitted retrospectively reviewed. Compared to US hospital trauma registry data.	2	506 (1 year)	No: retrospective data collection of admitted patients. Not trauma registry.
Uganda	Demyttenaere et al. 2009 [4]	Paper trauma registry forms completed for all trauma patients in emergency department.	1	3778 (1 year)	Yes
Uganda	Hsia et al. 2010 [11]	Paper trauma registry forms completed for all trauma patients in emergency department.	1	3750 (1 year)	No: same data set as Demyttenaere et al.’s paper.
Uganda	Kobusingye and Lett 2000 [14]	Paper trauma registry forms completed for all trauma patients in emergency department.	2	5210 (no study period available in paper)	No: no study period available to assess if same patients in Kobusingye et al. paper from 2002.
Uganda	Kobusingye et al. 2002 [13]	Paper trauma registry forms completed for all trauma patients in emergency department.	5 (citywide)	4359 (1 year)	Yes
Zambia	Seidenberg et al. 2014 [41]	Paper trauma registry forms completed for patients if they presented to the Surgical Emergency Centre with evidence of injury. Additional data collected on those brought in dead through the same Emergency Centre.	1	3498 (6 months)	Yes

**Table 2.** Assessment of quality of articles.

Author, Year	Data capture	Reported completeness of data (%)	Data collection staff	Trauma data collection methods	Methods to optimise data quality	Overall subjective assessment
Demyttenaere et al. 2009 [4]	Prospective	93.5	Not mentioned	Paper form	Not mentioned	Good
Haghparast-Bidgoli et al. 2013 [8]	Prospective	Not mentioned	Trained physicians	Validated questionnaire then data analysed using IBM SPSS Statistics	Trained physicians doing data collection	Good
Hashmi et al. 2013 [9]	Prospective	90	Trained personnel	Not mentioned	Data collection by trained personnel	Good
Hsia et al. 2010 [11]	Prospective	93	Doctors, nurses and clinical officers	Paper form then entered onto computer spreadsheet	Data checked by Senior Doctor	Moderate
Kobusingye and Lett 2000 [14]	Prospective	Not mentioned	Staff trained for 1 h	One page paper form then loaded onto Epi Info Version 6	Crosschecked with hospital registration book	Moderate
Kobusingye et al. 2002 [13]	Prospective	96.5	Doctors, nurses and clinical officers	One page paper form	Data checked by Senior Doctor	Good
Laing et al. 2014 [17]	Prospective	80	Trained physicians	Computer questionnaire then analysed using FileMaker Pro 11	Trained doctors	Good
Mehmood et al. 2013 [20]	Prospective	97	Trained research assistant	Paper form then analysed using Karachi Trauma Registry Software	Random checks of data collection by Principal Investigator	Good
Moini et al. 2000 [22]	Prospective	95	Trained physicians	Paper form then Epi Info then analysed using IBM SPSS	Trained physicians	Good
Nottidge et al. 2014 [25]	Prospective	Varied completeness of data collection	Not mentioned	Paper form then Epi Info	Not mentioned	Moderate-poor
Ordóñez et al. 2012 [29]	Prospective and retrospective	37.6	Full time staff for data recording	International Trauma Registry web-based form	Electronic retrieval from electronic notes	Good
Petroze et al. 2014 [32]	Prospective	Not mentioned	Trained data manager	Paper form then entered into Microsoft Access	Trained data manager	Good
Plummer et al. 2010 [33]	Prospective	Not mentioned	Not mentioned	Collected and transferred to Trauma! Software programme	Not mentioned	Moderate-poor
Rabbani and Moini 2007 [34]	Prospective	Not mentioned	Trained physicians	Not mentioned	Trained physicians	Moderate-poor
Roy et al. 2010 [37]	Prospective	95	Medical intern collecting data	Questionnaire then analysed using STATA	Dedicated intern collecting data	Good
Sabariah et al. 2008 [38]	Not mentioned	Not mentioned	Not mentioned	Not mentioned	Not mentioned	Moderate-poor
Samuel et al. 2010 [39]	Prospective	Not mentioned	Trained registry clerk 24 h/day	Double-sided registry form	Trained registry clerk 24 h/day	Moderate
Schuurman et al. 2011 [40]	Prospective	Varied: displayed as a table in the paper	Two trained researchers	Paper form	Two trained researchers	Good
Seidenberg et al. 2014 [41]	Prospective	Not mentioned	Trained staff 24 h/day	Registry questionnaire, then Cardiff Teleform	Trained staff 24 h/day and data collected twice daily when admitted	Good
Squyer et al. 2008 [42]	Retrospective	75	Not mentioned	Medical records reviewed from trauma patients	Not mentioned	Moderate
Wainiqolo et al. 2012 [43]	Not mentioned	Not mentioned	Research assistants and hospital nurses	Injury surveillance questionnaire	Research assistants	Moderate
Ward et al. 2010 [44]	Prospective and retrospective	Not mentioned	Trained medical records clerks	Not mentioned	Trained medical records clerks	Moderate
Zafar et al. 2002 [51]	Prospective	97	Trained researcher	Trauma paper form then electronic Trauma Registry v3.0	Trained researcher	Good

Table 3 displays the quantitative synthesis of the review. The median age of LMIC trauma victims in this analysis was 27 (IQR 25–29). The median percentage of trauma victims who were male was 75 (IQR 66–84). The median percentage of RTIs as a percentage of total injuries caused by trauma was 46 (IQR 21–71). The median percentage of penetrating injuries (stabbings and gunshots) as a percentage of total injuries caused by trauma was 10 (IQR 4–21). The median percentage of blunt force injuries as a percentage of total injuries caused by trauma was 1 (IQR 0–15). The median percentage of falls as a percentage of total injuries caused by trauma was 17 (IQR 8–31). We found four of the articles in the quantitative synthesis of the review used the “Kampala Trauma Score” [13] to calculate the severity of injuries in their patients. Other trauma scoring systems used included the “Abbreviated Injury Scale” (AIS), the “A Severity Characterization Of Trauma” (ASCOT) score, the Glasgow Coma Scale, the “Injury Severity Score”, the “Revised Trauma Score” and the “Trauma and Injury Severity Score”.

**Table 3.** Quantitative synthesis of systematic review.

Author, Year	Average age (years)	% Male	% Road traffic injuries (RTIs)	% Stabbing or gunshot	% Blunt force	% Fall	% Other cause	Trauma scoring system(s) used (see below for abbreviations)
Demyttenaere et al. 2009 [4]	26 (mean)	75	50	10	15	10	2	KTS
Haghparast-Bidgoli et al. 2013 [8]	31 (mean), 26 (median)	78	47	Not mentioned	Not mentioned	Not mentioned	Not mentioned	GCS, ISS
Hashmi et al. 2013 [9]	Most patients 26–35	87	59	19.6	0	5.6	9.1	GCS, ISS, RTS
Kobusingye et al. 2002 [13]	24 (mean)	73	50	16	0	13	–	KTS
Laing et al. 2014 [17]	28 (mean)	82	Not documented	40.5	54.7 (includes RTIs)	0	4.8	ISS
Mehmood et al. 2013 [20]	27 (mean)	72	33	7	0	37	16	GCS, ISS, RTS, TRISS
Nottidge et al. 2014 [25]	Most patients 20–39	74	“Most”	Not mentioned	Not mentioned	Not mentioned	Not mentioned	AIS. ISS
Ordóñez et al. 2012 [29]	31 (mean)	67	21	19.8	0	33.7	20.8	GCS, ISS, RTS
Petroze et al. 2014 [32]	30 (mean), 27 (median)	75	48	4	14	28	6	GCS
Rabbani and Moini 2007 [34]	28 (mean)	78	46	5.1	14.9	19	0	AIS, ASCOT, ISS, TRISS
Roy et al. 2010 [37]	30 (mean)	84	46	0	0	17	29	Not mentioned
Sabariah et al. 2008 [38]	Most patients 15–24	84	73	Not mentioned	Not mentioned	Not mentioned	Not mentioned	GCS, ISS
Samuel et al. 2010 [39]	26 (median)	76	43	0	0	13.5	29.6	Not mentioned
Schuurman et al. 2011 [40]	Most patients 20–39	75	22	22	16	0	0	AIS, ISS, KTS
Seidenberg et al. 2014 [41]	24 (median)	72	26	3.4	2.7	26.3	25.8	KTS
Ward et al. 2010 [44]	Most under 29	64	17	27.4	17	44	3.6	Not mentioned

Abbreviations of trauma scores: AIS: Abbreviated Injury Scale; ASCOT: A Severity Characterization Of Trauma; GCS: Glasgow Coma Scale; ISS: Injury Severity Score; KTS: Kampala Trauma Score; RTS: Revised Trauma Score; TRISS: Trauma and Injury Severity Score.

Table 4 displays the pre-hospital transfer methods and transfer times. Only a few of the articles reported this data. The median transfer time to hospital was 180 min with a large range of transfer times. Pre-hospital transfer methods varied largely between countries and between the articles. The median percentage of ambulance transfers as a percentage of total pre-hospital transfers was 6 (IQR 5–35). The median percentage of private vehicle transfers as a percentage of total pre-hospital transfers was 44 (IQR 0–52). Other less common methods of pre-hospital transfer included walking, taxi, public transport and police.

**Table 4.** Pre-hospital transfer times and methods review.

Author, Year	Pre-hospital transfer (PHT) time (minutes)	Ambulance (%)	Private Vehicle (%)	Walking (%)	Public transport (%)	Police (%)	Bike/ motorbike (%)	Taxi (%)	Other (%)
Hsia et al. 2010 [11]	Not mentioned	5	50	10	0	22	12	0	0
Kobusingye et al. 2002 [13]	66% patients within 60 min	–	–	–	–	–	–	–	–
Mehmood et al. 2013 [20]	81% patients within 360 min								
Nottidge et al. 2014 [25]	Not mentioned	0	98	0	0	0	0	0	2
Roy et al. 2010 [37]	Not mentioned	34.5	0	0	0	24.4	0	39.3	0
Samuel et al. 2010 [39]	201	15.4	43.8	14.5	12.4	7.8	0	0	0
Seidenberg et al. 2014 [41]	180	5.8	51.8	0	37.1	0	0	0	0
Squyer et al. 2008 [42]	Not mentioned	78.7	21.3	0	0	0	0	0	0
Zafar et al. 2002 [51]	98	6.4	0	0	0	0	0	0	93.5

## Discussion

This systematic review of published trauma registry data demonstrates that young, male, road traffic victims represent a large proportion of the LMIC trauma burden. These findings are consistent with a previous systematic analysis of the global burden of disease in young people, which found that RTIs accounted for the most disability-adjusted life years (DALYs) in young males aged 10–24 years [7].

Amongst the global population of all ages RTIs accounted for 75.5 million DALYs in 2010, an increase from 56.7 million in 1990 [23]. RTIs accounted for 53% more of the global burden of disease than tuberculosis in 2010 [23]. Despite this burden, the epidemic of injuries has been described as being “among the most neglected health problems of the late 20th century” [46] with relatively little research conducted into road safety injuries compared to other leading causes of disease [16]. Indeed, investment in injury has fallen behind investments in HIV/AIDS and reproductive health [7]. It is estimated that if injury mortality rates from all causes of injury in LMICs were reduced to those rates seen in high-income countries, over two million lives could be saved each year [15]. Financially, RTIs are estimated to cost LMICs 100 billion US dollars per year according to the World Bank [45], representing 1–3% of their gross national product (GNP) [36].

Improved road safety programmes can result in dramatic reductions in road traffic injury rates, as demonstrated in Australia where there was a 43.7% reduction in road traffic-related mortality following the introduction of road safety measures in 1990 [23]. Such safety measures, in combination with road safety education, are urgently required in LMICs [11, 16] and the health sector should champion these measures, as recommended by the World Health Organization [35]. RTIs can be reduced by enforcing speed limits, drink-driving laws, seat-belt laws and helmet use amongst motorcyclists [3].

The need to improve road safety globally has previously been highlighted but there has been limited action taken in LMICs [12]. This led to the initiation of the “Road Safety in 10 Countries Project” being initiated in 2012 [12]. This highly promising road safety project is predicted to save 10,310 lives over 5 years [6]. Positive potential side-effects of improved road safety are an increase in walking and cycling and a reduction in pollution [2].

The young men identified by this review as most affected by trauma are also often the family breadwinners in LMICs [47] and their death or disability from injury may drive these families into poverty [48]. Similarly the cost of care for injured young men can place unsustainable demands on families, especially in the context of underdeveloped social care and security systems [50].

This systematic review utilised data from trauma registries to determine the demographics of trauma patients in LMICs. In a scoping review of world trauma registries in 2012, publications from trauma registries were identified from 35 countries with the majority of publications from the US and Australia [27]. Trauma registries can be used as part of a trauma quality improvement programme [9, 29]. Implementation of trauma quality improvement programmes, which include trauma registries, has resulted in decreased mortality from trauma [9]. By identifying trends in injury, prevention strategies can be designed [4]. Trauma quality improvement programmes may also reduce overall hospital costs [5].

South Africa was identified to have a relatively high rate of penetrating injuries (including stabbings and gunshots). In the paper by Laing et al. they reported 40.5% of their injuries to be penetrating [17]. Jamaica also had a relatively high rate of penetrating trauma with 27.4% of the injuries in the paper by Ward et al. attributed to penetrating trauma [44]. This relatively high rate of violent trauma in these countries should be addressed by the local governments. Laing et al. discuss the fact that there is a high rate of interpersonal violence in South Africa [17] and Ward et al. explain that the “Violence Prevention Programme” was set up in Jamaica in 2004 to address the growing problem [44].

Time to hospital varied largely between countries and only a few of the trauma registry articles in this review, contained this information. In LMIC trauma registries in this review pre-hospital transfer times were long and the availability of ambulance transfers was limited. Long pre-hospital transfer times may be associated with worse outcomes [39]. This is an issue that needs addressing by local governments.

The quality of articles analysed in this review was variable. In a review by O’Reilly et al. they devised a tool to analyse data from trauma registries, which they named the “Trauma Registry Assessment Tool” [28]. This tool helps to assess the physical resources, human resources and processes of a trauma registry and is displayed in Table 1 of their paper [28]. We used the tool to assess the overall quality of articles we analysed. Table 2 displays our assessment of article quality using this assessment tool. Most articles reported prospective data but completeness of data collection was often not reported. Most trauma registries initially collected data on paper forms and then transferred this information to computer. We would like to propose that trauma registries report their data in the format of the “Trauma Registry Assessment Tool”. This would ensure that articles from trauma registries would be of a consistently high standard and that all important data is published. By presenting the data in this way it would allow funding bodies and governments to identify the areas of greatest need of investment and support. Trauma registries are expensive to run and therefore have an ethical obligation to publish data in an easy-to-read and consistent format so that their cost can be justified.

A limitation of this systematic review is that it only includes data from trauma registries and linked data may better estimate the age, gender and mechanism of injury in LMIC trauma patients. Additionally, trauma registry data may suffer from a decreased capture rate of data because busy clinicians may not have the time to record every trauma episode [13, 42]. Trauma registry data will only capture the data of injured patients who attend hospitals with trauma registries and will miss those patients who have their injuries treated in the community or die before reaching hospital [14, 40].

In summary, this trauma registry study has identified that the young, male population is most affected by trauma in LMICs and 46% of all injuries were road traffic injuries. This information can be used by local and national governments to support the case for increased investment in road safety measures and other strategies targeted at injury prevention in this population group.

## Conflicts of interest

OB and GGJ declare no conflicts of interest.

CBDL and CEG are both Lancet Commissioners on Global Surgery but declare no conflicts of interest related to this work.

## References

[1] Anon (2014) The World Bank. Data: country and lending groups. World Bank Retrieved August 30, 2014 (http://data.worldbank.org/about/country-and-lending-groups).

[2] Bliss T, Raffo V (2013) Improving global road safety: Towards equitable and sustainable development, guidelines for country road safety engagement. World Bank Retrieved Feb 22, 2015 (https://openknowledge.worldbank.org/handle/10986/17627).

[3] Chisholm D, Naci H, Hyder AA (2012) Cost effectiveness of strategies to combat road traffic injuries in Sub-Saharan Africa and South East Asia: mathematical modelling study. BMJ 344, e612. 22389340 10.1136/bmj.e612 PMC3292520

[4] Demyttenaere SV et al. (2009) Injury in Kampala, Uganda: 6 Years Later. Can J Surg 52(5), E146–E150. 19865544 PMC2769114

[5] DiRusso S et al. (2001) Preparation and achievement of American college of Surgeons level I trauma verification raises hospital performance and improves patient outcome. J Trauma 51, 294–300. 11493787 10.1097/00005373-200108000-00011

[6] Esperato A, Bishai D, Hyder AA (2012) Projecting the health and economic impact of road safety initiatives: a case study of a multi-country project. Traffic Inj Prev 13(Suppl 1), 82–89. 22414132 10.1080/15389588.2011.647138

[7] Gore FM et al. (2011) Global burden of disease in young people aged 10–24 years: a systematic analysis. Lancet 377(9783), 2093–2102. 21652063 10.1016/S0140-6736(11)60512-6

[8] Haghparast-Bidgoli H, Saadat S, Bogg L, Yarmohammadian MH, Hasselberg M(2013) Factors affecting hospital length of stay and hospital charges associated with road traffic-related injuries in Iran. BMC Health Serv Res 13(1), 281. 23875993 10.1186/1472-6963-13-281 PMC3726419

[9] Hashmi ZG et al. (2013) Hospital-based trauma quality improvement initiatives: first step toward improving trauma outcomes in the developing world. J Trauma Acute Care Surg 75(1), 60–68. 23778440 10.1097/TA.0b013e31829880a0

[10] Higgins J, Deeks J (2008) Chapter 7: selecting studies and collecting data, in Cochrane handbook of systematic reviews of interventions. Higgins JPT, Green S, Editors Chicester (UK), John Wiley & Sons.

[11] Hsia RY et al. (2010) Epidemiology of injuries presenting to the national hospital in Kampala, Uganda: implications for research and policy. Int J Emerg Med 3(3), 165–172. 21031040 10.1007/s12245-010-0200-1 PMC2926872

[12] Hyder AA et al. (2012) Addressing the implementation gap in global road safety: exploring features of an effective response and introducing a 10-country program. Am J Public Health 102(6), 1061–1067. 22515864 10.2105/AJPH.2011.300563 PMC3483956

[13] Kobusingye OC, Guwatudde D, Owor G, Lett RR (2002) Citywide trauma experience in Kampala, Uganda: a call for intervention. Inj Prev 8(2), 133–136. 12120832 10.1136/ip.8.2.133 PMC1730841

[14] Kobusingye OC, Lett RR (2000) Hospital-based trauma registries in Uganda. J Trauma 48(3), 498–502. 10744292 10.1097/00005373-200003000-00022

[15] Kotagal M et al. (2014) Health and economic benefits of improved injury prevention and trauma care worldwide. PLoS One 9(3), e91862. 24626472 10.1371/journal.pone.0091862 PMC3953529

[16] Lagarde E (2007) Road traffic injury is an escalating burden in Africa and deserves proportionate research efforts. PLoS Med 4(6), e170. 17593893 10.1371/journal.pmed.0040170 PMC1896192

[17] Laing GL, Bruce JL, Aldous C, Clarke DL (2014) The design, construction and implementation of a computerised trauma registry in a developing South African metropolitan trauma service. Injury 45(1), 3–8. 23827395 10.1016/j.injury.2013.05.013

[18] Lozano R et al. (2012) Global and regional mortality from 235 causes of death for 20 age groups in 1990 and 2010: a systematic analysis for the Global Burden of Disease Study 2010. Lancet 380(9859), 2095–2128. 23245604 10.1016/S0140-6736(12)61728-0 PMC10790329

[19] Mathers CD, Loncar D (2006) Projections of global mortality and burden of disease from 2002 to 2030. PLoS Med 3(11), e442. 17132052 10.1371/journal.pmed.0030442 PMC1664601

[20] Mehmood A, Razzak JA, Kabir S, Mackenzie EJ, Hyder AA (2013) Development and pilot implementation of a locally developed trauma registry: lessons learnt in a low-income country. BMC Emerg Med 13(1), 4. 23517344 10.1186/1471-227X-13-4 PMC3606628

[21] Moher D, Liberati A, Tetzlaff J, Altman DG (2010) Preferred reporting items for systematic reviews and meta-analyses: the PRISMA statement. Int J Surg 8(5), 336–341. 20171303 10.1016/j.ijsu.2010.02.007

[22] Moini M, Rezaishiraz H, Zafarghandi MR (2000) characteristics and outcome of injured patients treated in urban trauma centers in Iran. J Trauma 48(3), 503–507. 10744293 10.1097/00005373-200003000-00023

[23] Murray CJL et al. (2012) Disability-adjusted life years (DALYs) for 291 diseases and injuries in 21 regions, 1990–2010: a systematic analysis for the Global Burden of Disease Study 2010. Lancet 380(9859), 2197–2223. 23245608 10.1016/S0140-6736(12)61689-4

[24] Norton R, Kobusingye O (2013) Injuries. NEJM 368(18), 1723–1730. 23635052 10.1056/NEJMra1109343

[25] Nottidge TE, Dim M, Udoinyang CI, Udoh IA (2014) The Uyo Trauma Registry-developed for sustainable audit of trauma care and cause in Nigeria. Trop Doct 44(1), 14–18. 24231684 10.1177/0049475513512632

[26] Nwomeh BC, Lowell W, Kable R, Haley K, Ameh EA (2006) History and development of trauma registry: lessons from developed to developing countries. World J Emerg Surg 1, 32. 17076896 10.1186/1749-7922-1-32 PMC1635421

[27] O’Reilly GM, Cameron PA, Joshipura M (2012) Global trauma registry mapping: a scoping review. Injury 43(7), 1148–1153. 22483995 10.1016/j.injury.2012.03.003

[28] O’Reilly GM, Joshipura M, Cameron PA, Gruen R (2013) Trauma registries in developing countries: a review of the published experience. Injury 44(6), 713–721. 23473265 10.1016/j.injury.2013.02.003

[29] Ordóñez CA et al. (2012) Experience of two first level hospitals in the southwest region of Colombia on the implementation of the Panamerican Trauma Society International Trauma Registry. Rev Col Bras Cir 39(4), 255–262. 22936222 10.1590/s0100-69912012000400003

[30] Patton GC et al. (2009) Global patterns of mortality in young people: a systematic analysis of population health data. Lancet 374(9693), 881–892. 19748397 10.1016/S0140-6736(09)60741-8

[31] Peden M, McGee K, Krug E (2002) Injury: a leading cause of the global burden of disease, 2000. Geneva, World Health Organization.

[32] Petroze RT et al. (2014) Infectious outcomes assessment for health system strengthening in low-resource settings: the novel use of a trauma registry in Rwanda. Surg Infect 15(4), 382–386. 10.1089/sur.2013.146 PMC4135322 24828195

[33] Plummer J et al. (2010) Trauma: the burden of a preventable problem trauma. West Indian Med J 59(1), 26–28. 20931909

[34] Rabbani A, Moini M (2007) Application of “Trauma and Injury Severity Score” and “A Severity Characterization of Trauma” score to trauma patients in a setting different from “Major Trauma Outcome Study”. Arch Iran Med 10(3), 383–386. 17604479

[35] Racioppi F, Eriksson L, Tingvall C, Villaveces A (2004) Preventing road traffic injury: a public health perspective for Europe. WHO Retrieved July 15, 2014 (http://www.euro.who.int/__data/assets/pdf_file/0003/87564/E82659.pdf).

[36] Raffo V, Bliss T, Shotten M, Sleet D, Blanchard C (2013) Case study: The Argentina Road Safety Project: lessons learned for the decade of action for road safety, 2011–2020. Glob Health Promot 20(4 Suppl), 20–36. 24722740 10.1177/1757975913502690 PMC6784537

[37] Roy N et al. (2010) Where there are no emergency medical services-prehospital care for the injured in Mumbai, India. Prehosp Disaster Med 25(2), 145–151. 20467994 10.1017/s1049023x00007883

[38] Sabariah F, Ramesh N, Mahathar A (2008) National Trauma Database (NTrD) – improving trauma care: first year report. Med J Malaysia 63, 45–49. 19227673

[39] Samuel JC et al. (2010) Hospital-based injury data in Malawi: strategies for data collection and feasibility of trauma scoring tools. Trop Doct 40(2), 98–99. 20305105 10.1258/td.2009.090009 PMC3290406

[40] Schuurman N et al. (2011) collecting injury surveillance data in low- and middle-income countries: the Cape Town Trauma Registry pilot. Glob Public Hlth 6(8), 874–889. 10.1080/17441692.2010.516268 20938854

[41] Seidenberg P et al. (2014) epidemiology of injuries, outcomes, and hospital resource utilisation at a tertiary teaching hospital in Lusaka, Zambia. Afr J Emerg Med 4(3), 115–122.

[42] Squyer E et al. (2008) comparison of trauma mortality between two hospitals in Turkey to one trauma center in the US. Eur J Emerg Med 15(4), 209–213. 19078816 10.1097/MEJ.0b013e3283034232

[43] Wainiqolo I et al. (2012) A profile of injury in Fiji: findings from a population-based injury surveillance system (TRIP-10). BMC Publ Health 12(1), 1074. 10.1186/1471-2458-12-1074 PMC3540002 23234597

[44] Ward E et al. (2010) The Jamaica Injury Surveillance System: a profile of the intentional and unintentional injuries in Jamaican hospitals. West Indian Med J 59(876), 7–13. 20931906

[45] World Bank (2014) World Bank. Roads & HIghways: Road Safety. Retrieved August 30, 2014 (http://www.worldbank.org/transport/roads/safety.htm).

[46] World Health Organization (1996) Investing in Health Research and Development. Retrieved Aug 20, 2014 (http://whqlibdoc.who.int/hq/1996/TDR_GEN_96.2.pdf).

[47] World Health Organization (2004) World report on road traffic injury prevention. Geneva, World Health Organization Retrieved July 15, 2014 (http://www.who.int/violence_injury_prevention/publications/road_traffic/world_report/summary_en_rev.pdf).

[48] World Health Organization (2009) Global status report on road safety: time for action. Geneva, World Health Organization Retrieved July 28, 2014 (www.who.int/violence_).

[49] World Health Organization (2010) Injuries and violence: the facts. Retrieved July 25, 2014 (http://whqlibdoc.who.int/publications/2010/9789241599375_eng.pdf).

[50] Young S (2014) Orthopaedic trauma surgery in low-income countries. Follow-up, infections and HIV. Acta Orthop 85(S356), 1–32. 25052728 10.3109/17453674.2014.937924

[51] Zafar H, Rehmani R, Raja AJ, Ali A, Ahmed M (2002) Registry based trauma outcome: perspective of a developing country. Emerg Med J 19(5), 391–394. 12204982 10.1136/emj.19.5.391 PMC1725962

